# Transcriptome analysis of alternative splicing in peanut (*Arachis hypogaea* L.)

**DOI:** 10.1186/s12870-018-1339-9

**Published:** 2018-07-04

**Authors:** Jian Ruan, Feng Guo, Yingying Wang, Xinguo Li, Shubo Wan, Lei Shan, Zhenying Peng

**Affiliations:** 1 College of Life Science, Shandong University, Jinan, China; 2 Bio-Tech Research Center, Shandong Academy of Agricultural Science/Shandong Provincial Key Laboratory of Genetic Improvement, Ecology and Physiology of Crops, Jinan, China

**Keywords:** *Arachis hypogaea* L., Alternative splicing, Transcriptome analysis, Organ-specific expression, Fatty acid metabolism

## Abstract

**Background:**

Alternative splicing (AS) represents a mechanism widely used by eukaryotes for the post-transcriptional regulation of genes. The detailed exploration of AS in peanut has not been documented.

**Results:**

The strand-specific RNA-Seq technique was exploited to characterize the distribution of AS in the four samples of peanut (FH1-seed1, FH1-seed2, FH1-root and FH1-leaf). AS was detected as affecting around 37.2% of the full set of multi-exon genes. Some of these genes experienced AS throughout the plant, while in the case of others, the effect was organ-specific. Overall, AS was more frequent in the seed than in either the root or leaf. The predominant form of AS was intron retention, and AS in transcription start site and transcription terminal site were commonly identified in all the four samples. It is interesting that in genes affected by AS, the majority experienced only a single type of event. Not all of the in silico predicted transcripts appeared to be translated, implying that these are either degraded or sequestered away from the translation machinery. With respect to genes involved in fatty acid metabolism, about 61.6% were shown to experience AS.

**Conclusion:**

Our report contributes significantly in AS analysis of peanut genes in general, and these results have not been mentioned before. The specific functions of different AS forms need further investigation.

**Electronic supplementary material:**

The online version of this article (10.1186/s12870-018-1339-9) contains supplementary material, which is available to authorized users.

## Background

In eukaryotes, AS, a process in which more than one transcript is produced from a single coding sequence, has evolved as a ubiquitous mode of post-transcriptional gene regulation [1, 2]. Defective AS has been associated with a number of clinical conditions [3, 4], genetic diseases [5–7] and aging [8]. In plants, AS has been shown to regulate growth, development, signal transduction, flowering, circadian clock function and the response to various environmental cues [9–13], as well as being associated with speciation [14–16]. Five forms of AS have been recognized, namely transcription start site (TSS), transcription terminal site (TTS), exon skipping (ES), intron retention (IR), alternative exon ends (5′, 3′, or both; AE) [17].

High throughput sequencing data sets provide a major opportunity for investigating the genome-wide distribution of AS. The impression to date is that the extent of AS increases with both organ and species complexity [18, 19]. In animal genomes the proportion of genes harboring intron(s) which experience AS lies in the range 20–95% [20–24], while the equivalent proportion in plant genomes is variable greatly [25–33]. In both animals and yeast, ES is the most prevalent form of AS, while IR is the least common [22, 24, 34, 35]. In contrast, in plants, most AS events involve IR, with the relative frequency of the five forms of AS differing across the monocotyledonous/dicotyledonous divide [25, 36–38]. Approximately 60–75% of AS events result in changes to the binding property, phosphorylation status, stability, intracellular localization, enzymatic activity or signaling activity of the gene product [1, 38–40]. Many AS-generated foreshortened transcripts are processed by nonsense-mediated decay or are regulated by microRNAs (miRNAs) [41–44]. At least 13% of intron-containing genes in *Arabidopsis thaliana* are potentially regulated by nonsense-mediated decay [45].

The peanut (*Arachis hypogaea* L*.*), a leading oil and protein crop, is an allotetraploid (2*n* = 4*×* = 40) developed from a hybrid between the two diploid wild species *A. duranensis* and *A. ipaensis*. Sequencing of these two progenitor species has generated coverage of about 96% of the cultivated peanut genome [46], thereby providing a firm basis for genetic investigations. As yet, the genome-wide occurrence of AS in peanut has not been explored. Here, the strand-specific RNA-Seq approach has been used to characterize the occurrence of AS in two distinct developmental stages of the seed, and in the seedling root and leaf of a leading peanut cultivar.

## Results

### Sequencing of the four organ-provenance libraries from peanut

Four samples (FH1-seed1, FH1-seed2, FH1-root and FH1-leaf) from peanut have been prepared for strand-specific RNA-Seq. The RNA-Seq platform realized 51.3 × 10^9^ bases, for which the Q30 value was > 88.90% and the range in GC content 44.48–50.48% (Additional file 1: Table S1). After editing, the remaining high quality sequence was represented by 27.25 × 10^9^ bases in FH1-seed1 and FH1-seed2 combined, 12.42 × 10^9^ bases in FH1-root and 11.67 × 10^9^ bases in FH1-leaf. The raw sequence data have been submitted to the NCBI BioProject database under accession number PRJNA354652. When the clean reads were aligned with the genomic sequences of *A. duranensis* and *A. ipaensis*, the matching ratio ranged from 79.91 to 87.49% (Additional file 2: Table S2); the highest ratio was associated with the FH1-leaf library, but the number of unique mapped reads was the lowest in this library. In all four libraries, the reads mapped more frequently to the ‘+’ rather than to the ‘-’ strand.

### The peanut transcriptome

**Fig. 1:**
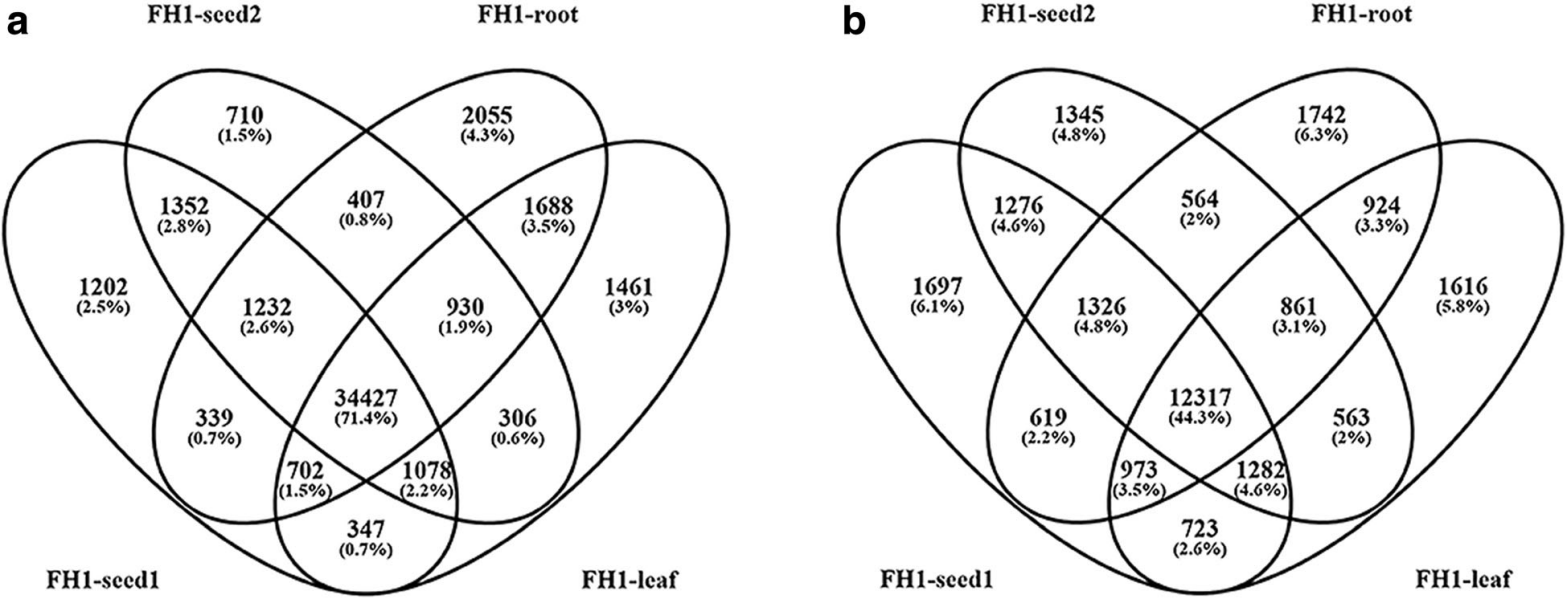
Venn diagram depicting the number of the expressed genes and AS genes in the four peanut organs. **a**, Venn diagram of the expressed genes; **b**, Venn diagram of the AS genes

The total 431,596 unique transcripts were pieced together in the four samples, 107,102 from FH1-seed1, 110,005 from FH1-seed2, 109,210 from FH1-root, and 105,279 from FH1-leaf, respectively (Table 1). Transcripts were detected for 54,047 genes (Table 2), which represents 68.8% of combined number of genes present in the two wild progenitors [47]. Following the application of fragments per kilobase of exon per million fragments mapped (FPKM) threshold of 0.1, the number of genes detected was reduced to 48,236 (Table 2). These genes broke down into 40,679 from FH1-seed1, 40,442 from FH1-seed2, 41,780 from FH1-root and 40,939 from FH1-leaf. The transcription of 34,427 of these genes was detected in all four libraries, leaving 1202 specific to FH1-seed1, 710 to FH1-seed2, 2055 to FH1-root and 1461 to FH1-leaf (Fig. 1a).

**Table 1:** Statistics of the number of the expressed genes and transcripts in the four samples from different peanut organs

Sample ID	Number of transcripts (with FPKM≥0.1)	Expressed Genes (with FPKM≥0.1)	Average number of transcripts per gene
FH1-seed1	107,102	40,679	2.63
FH1-seed2	110,005	40,442	2.72
FH1-root	109,210	41,780	2.61
FH1-leaf	105,279	40,939	2.57

**Table 2:** Statistics of the number of expressed genes and AS genes in the four samples from different peanut organs

Sample ID	Total detected genes (with count number ≥ 1)	Expressed Genes (with FPKM≥0.1)	AS genes (with FPKM≥0.1)	AS genes / Expressed genes (%)
FH1-seed1	49,298	40,679	20,213	49.69%
FH1-seed2	46,343	40,442	19,534	48.30%
FH1-root	46,982	41,780	19,326	46.26%
FH1-leaf	48,548	40,939	19,259	47.04%
Total	54,047	48,236	27,829	57.69%

### AS in the peanut transcriptome

Applying the FPKM threshold of 0.1 to the set of 48,236 transcribed genes revealed 27,829 genes as showing evidence of AS (Table 2): this figure represents 57.69% of the transcriptome and 37.2% of the full set of multi-exon genes predicted from the genome sequence. The four libraries harbored, respectively, 20,213 (FH1-seed1), 19,534 (FH1-seed2), 19,326 (FH1-root) and 19,259 (FH1-leaf) genes affected by AS. A total of 12,317 of the AS genes was represented in all four of the libraries, while the number of library-specific AS genes was, respectively, 1697, 1345, 1742 and 1616 (Fig. 1b). A total of 4318 genes were confined to the two libraries prepared from developing seed. AS events were overall more frequent in the seed than in the root or leaf (Table 3), and the number of splicing isoforms detected in seed was also more than that in root and leaf (Additional file 3: Table S3), implying a particular importance for AS in seed development.

**Table 3:** Statistics of the number of AS events and AS types in the four samples from different peanut organs

AS types	FH1-seed1		FH1-seed2		FH1-root		FH1-leaf	
	AS Events	Percentage	AS Events	Percentage	AS Events	Percentage	AS Events	Percentage
TSS	17,584	19.01%	16,980	19.85%	16,572	20.78%	16,632	19.97%
TTS	14,523	15.70%	13,657	15.96%	13,758	17.25%	13,717	16.47%
ES	10,891	11.78%	12,124	14.17%	10,423	13.07%	9726	11.68%
IR	30,931	33.45%	24,788	28.97%	21,408	26.84%	25,865	31.05%
AE	18,554	20.06%	18,013	21.05%	17,602	22.07%	17,350	20.83%
Total	92,483	100.00%	85,562	100.00%	79,763	100.00%	83,290	100.00%

The number of AS events detected in FH1-seed1 was 92,483, in FH1-seed2 85,562, in FH1-root 79,763 and in FH1-leaf 83,290 (Table 3). According to [17], five distinct types of AS event can be recognized, namely TSS, TTS, ES, IR, and AE (Table 2). The most frequent AS event observed in peanut (26.84–33.45%) involved IR, and the least frequent (11.68–14.17%) involved ES. AE events accounted for, respectively, 20.06–22.07%. These estimates agree with previous studies in other plants. TSS and TTS events accounted for, respectively, 19.01–20.78% and 15.70–17.25% respectively.

TSS and TTS events were commonly identified in the four samples. Within the set of FH1-seed, 17,584 TSS events affected 14,314 genes, 14,523 TTS events affected 12,680 genes, the number of TSS and TTS events generated per gene is 1.22 and 1.15 respectively (Table 4). Within the four samples, the number of TSS and TTS events generated per gene is 1.18–1.22 and 1.12–1.15 respectively (Table 4), the number of TSS events generated per gene is more than that of TTS events.

**Table 4:** Statistics of the number of TSS and TTS events occurred in the four samples of peanut different organs

	Sample ID	AS events	AS genes	Events/gene
TSS	FH1-seed1	17,584	14,431	1.22
	FH1-seed2	16,980	14,250	1.19
	FH1-root	16,572	14,071	1.18
	FH1-leaf	16,632	13,829	1.20
TTS	FH1-seed1	14,523	12,680	1.15
	FH1-seed2	13,657	12,142	1.12
	FH1-root	13,758	12,338	1.12
	FH1-leaf	13,717	12,036	1.14

An ES ‘event’ was defined as a pairing between ‘ON’ and ‘OFF’, occurring at the same exon and with the same flanking introns. The same exon or intron may be involved in multiple exon skipping (MSKIP) events, so the estimated number of ES events is more than its real number. Among the ES events, there was a higher number of SKIP_OFF than SKIP_ON events (Table 5). An IR ‘event’ was also defined as a pairing (IR_ON, IR_OFF). It is interesting that the number of IR_ON events were more than that of IR_OFF ones (Table 6).

**Table 5:** Comparison of different types of ES events occurred in the four samples of peanut different organs

AS types	FH1-seed1		FH1-seed2		FH1-root		FH1-leaf	
	AS Events	Percentage	AS Events	Percentage	AS Events	Percentage	AS Events	Percentage
SKIP_OFF	3189	29.28%	3546	29.25%	3003	28.81%	2840	29.20%
SKIP_ON	2945	27.04%	3316	27.35%	2797	26.83%	2583	26.56%
XSKIP_OFF	1759	16.15%	1832	15.11%	1680	16.12%	1586	16.31%
XSKIP_ON	1419	13.03%	1515	12.50%	1399	13.42%	1296	13.33%
MSKIP_OFF	593	5.44%	695	5.73%	548	5.26%	527	5.42%
MSKIP_ON	426	3.91%	554	4.57%	396	3.80%	372	3.82%
XMSKIP_OFF	341	3.13%	396	3.27%	364	3.49%	298	3.06%
XMSKIP_ON	219	2.01%	270	2.23%	236	2.26%	224	2.30%
Total	10,891	100.00%	12,124	100.00%	10,423	100.00%	9726	100.00%

*SKIP* Skipped exon (SKIP_ON, SKIP_OFF pair), *XSKIP* Approximate SKIP (XSKIP_ON, XSKIP_OFF pair), *MSKIP* Multi-exon SKIP (MSKIP_ON, MSKIP_OFF pair), *XMSKIP* Approximate MSKIP (XMSKIP_ON, XMSKIP_OFF pair)

**Table 6:** Comparison of different types of IR events occurred in the four samples of peanut different organs

AS types	FH1-seed1		FH1-seed2		FH1-root		FH1-leaf	
	AS Events	Percentage	AS Events	Percentage	AS Events	Percentage	AS Events	Percentage
IR_OFF	9331	30.17%	7584	30.60%	6573	30.70%	7986	30.88%
IR_ON	10,261	33.17%	8451	34.09%	7331	34.24%	8802	34.03%
XIR_OFF	2806	9.07%	2195	8.86%	2028	9.47%	2350	9.09%
XIR_ON	3400	10.99%	2803	11.31%	2516	11.75%	2905	11.23%
MIR_OFF	1700	5.50%	1179	4.76%	892	4.17%	1350	5.22%
MIR_ON	2133	6.90%	1529	6.17%	1191	5.56%	1720	6.65%
XMIR_OFF	526	1.70%	402	1.62%	344	1.61%	296	1.14%
XMIR_ON	774	2.50%	645	2.60%	533	2.49%	456	1.76%
Total	30,931	100.00%	24,788	100.00%	21,408	100.00%	25,865	100.00%

*IR* Intron retention (IR_ON, IR_OFF pair), *XIR* Approximate IR (XIR_ON, XIR_OFF pair), *MIR* Multi-IR (MIR_ON, MIR_OFF pair), *XMIR* Approximate MIR (XMIR_ON, XMIR_OFF pair)

**Fig. 2:**
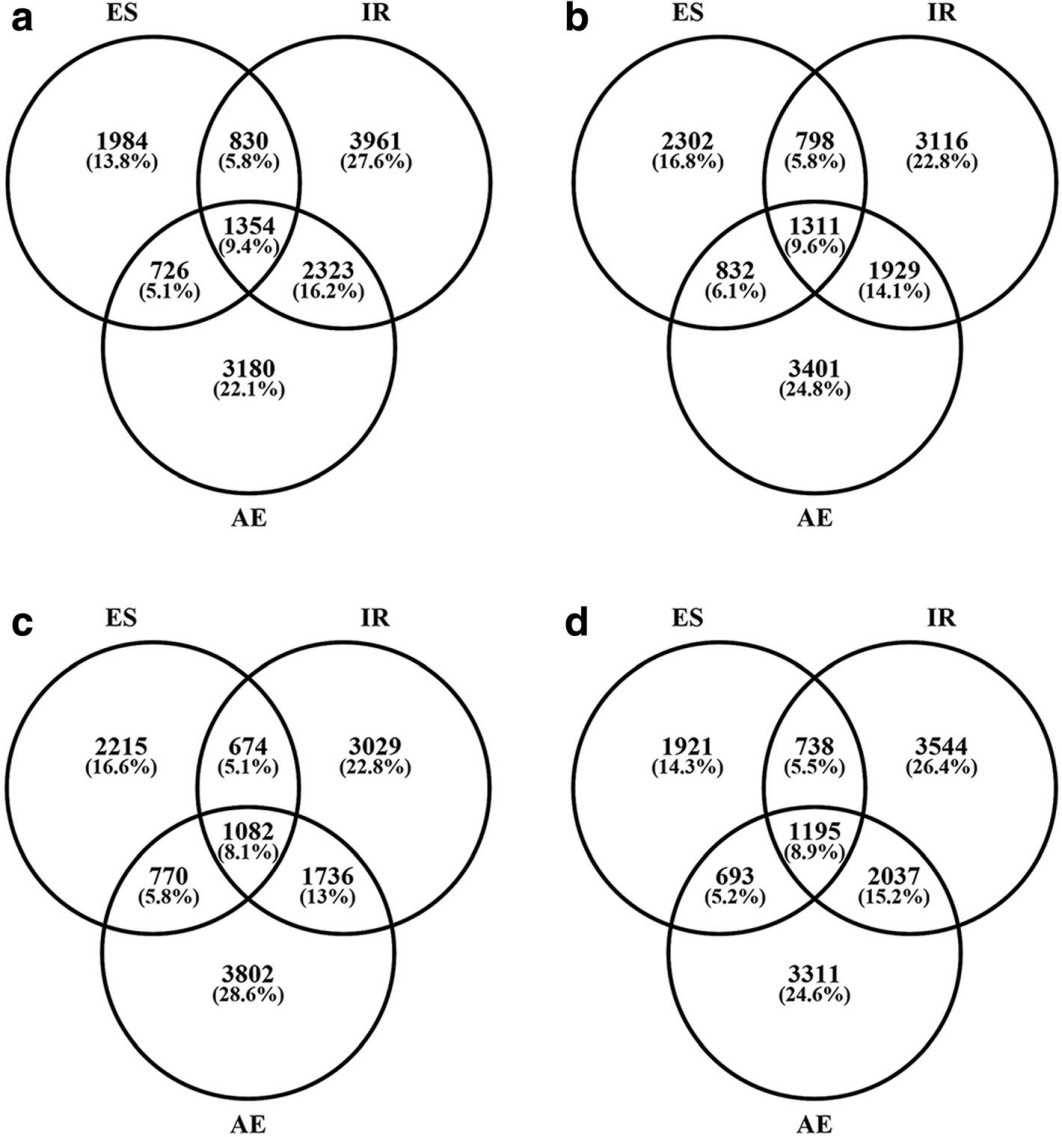
Venn diagram depicting the gene number of the three types of AS events occurred in the four peanut organs. **a**, FH1-seed1; **b**, FH1-seed2; **c**, FH1-root; **d**, FH1-leaf

Within the set of FH1-seed AS genes, 10,891 ES events affected 4894 genes, 30,931 IR events affected 8468 genes and 18,554 AE events affected 7583 genes (Table 2, Fig. 2a). The number of genes affected exclusively by ES, IR and AE was, respectively, 1984, 3961 and 3180. Thus, altogether, 9125 (63.55%) genes experienced only a single AS type, 3879 (27.02%) genes experienced two AS types, while only 1354 (9.43%) experienced three types. And also, the AS genes experienced simultaneously IR & AE were more than that experienced simultaneously IR & ES, and AE & ES. The transcriptomes represented in the other three libraries displayed a similar distribution of AS events (Fig. 2b-d). Overall, in genes affected by AS, the majority experienced only a single type of event.

Most of the AS genes produced a predominant transcript with a considerable higher level of expression which was detected in all four libraries, along with additional ones occurring at a substantially lower frequency (Additional file 4: Table S4). For example, Aradu.000JC generated four transcripts, two of which were detected in all four libraries, one in FH1-seed1 and FH1-seed2 (but not in either FH1-root or FH1-leaf), and the fourth in FH1-root and FH1-leaf (but not in either FH1-seed1 or FH1-seed2). In a second example, Araip.GAW68 generated 20 transcripts, five of which were represented in all four libraries, only one represented in three libraries, seven ones appeared in two libraries, and seven ones appeared in one library.

### Statistical analysis of the exon number of the peanut AS gene

**Fig. 3:**
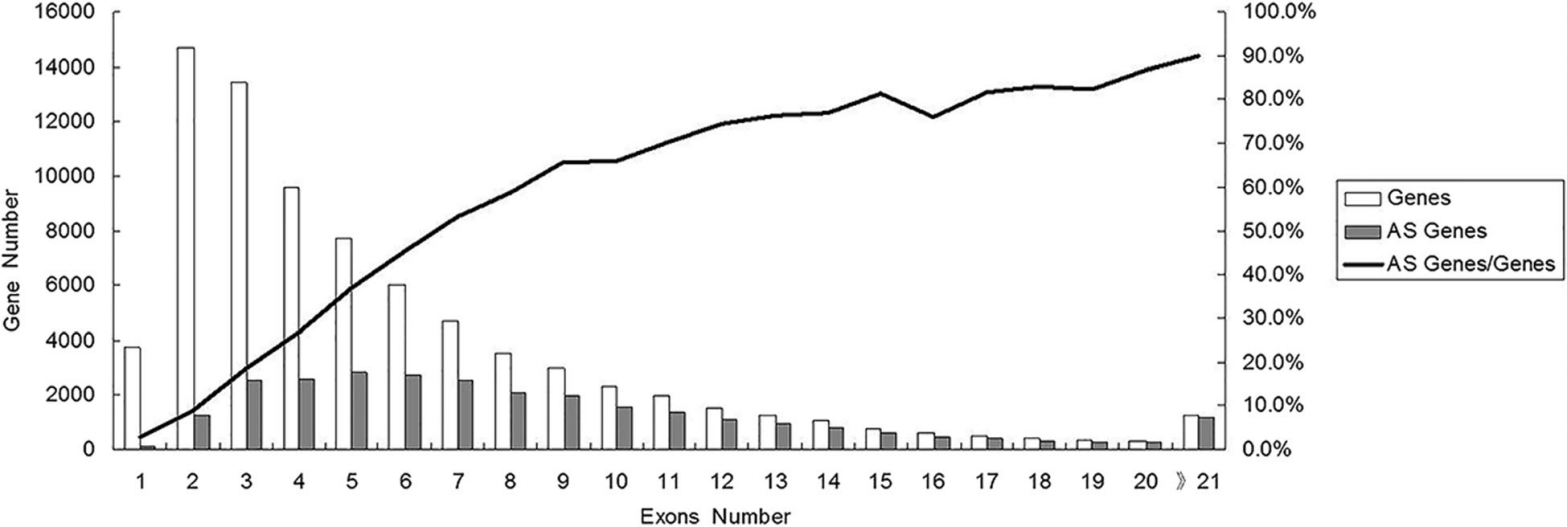
Comparison of the exon number between the total genes and AS genes in peanut genome. The number in X axes indicated the number of exons contained in the genes, the left Y axes indicated the number of the genes containing these exons, and the right Y axes indicated the ratio of AS genes/total genes with the same number of exons

Cultivated peanut contain two subgenomes (A and B). Although the genome sequencing of cultivated peanut has not been completed, the genome sequencing of two progenitor species of peanut (*A. duranensis* and *A. ipaensis*) has been finished, which contain 78,574 genes (36,734 and 41,840 genes, respectively) [46]. So we use the two wild peanut genomes as reference to analyze our transcriptome data. We analyzed the exon number of 78,574 genes (Fig. 3), and found that the number of intron-less genes were 3740, accounting for 4.76% of all genes; the number of genes containing 2 or more exons accounted for about 95% of the total genes. 47.26% genes contain ≥5 exons and 15.56% genes contain ≥10 exons. We analyzed exon number of 27,829 AS genes (Fig. 3). Results showed that the exon number of AS genes distributed mainly from 3 to 10, which accounted for 67.33% of all AS genes. And that, the occurrence rate of AS genes increased along with the exon number increasing. This was consistency with other reports [48].

### GO annotation and GO classification analysis of the peanut AS genes

**Fig. 4:**
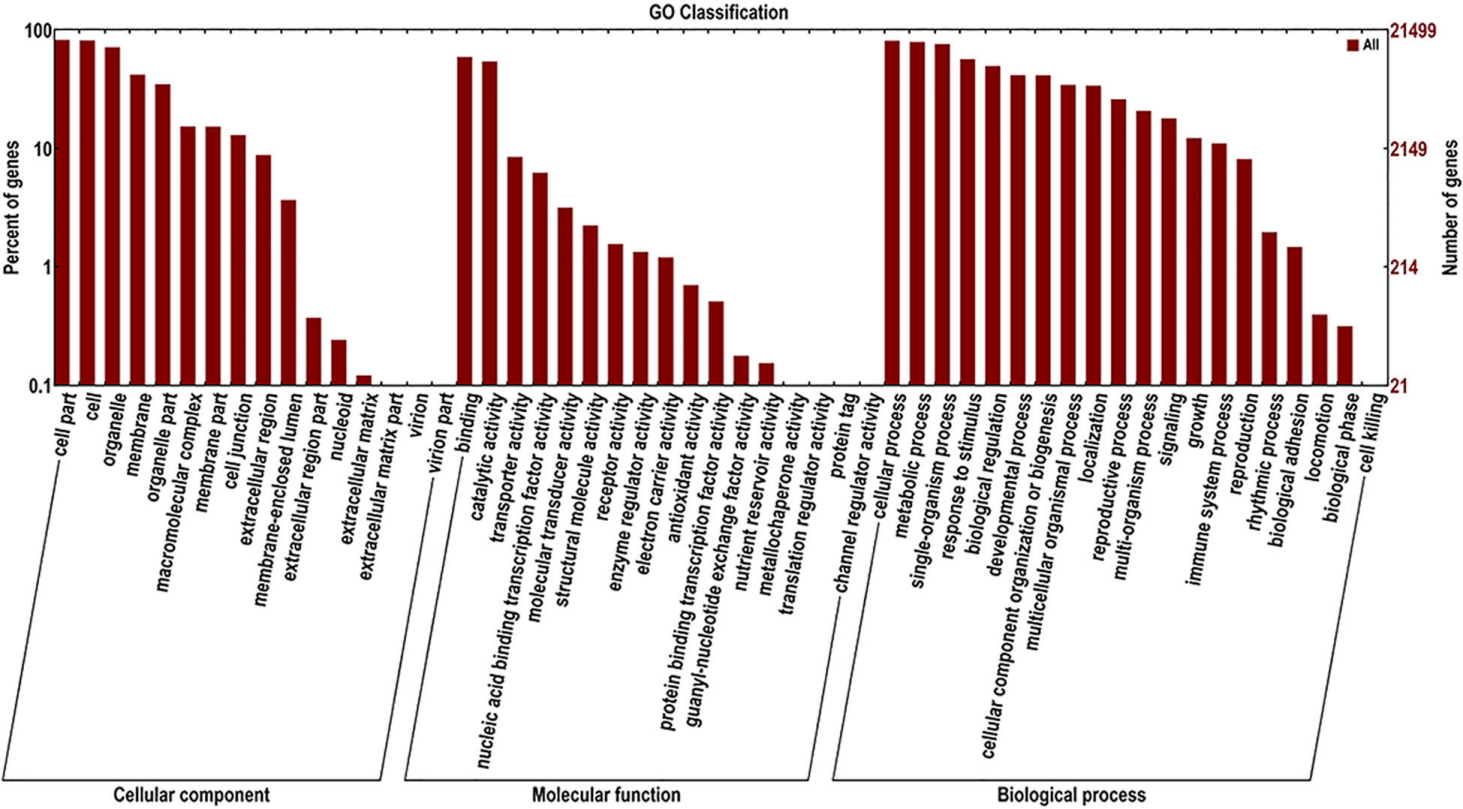
GO annotation and GO classification of the peanut AS genes

We conducted the gene function annotation to several different databases, such as NCBI non-redundant protein sequences (Nr), Clusters of Orthologous Groups of proteins (KOG/COG), Kyoto Encyclopedia of Genes and Genomes (KEGG) and Gene Ontology (GO). Here we analyzed in detail the GO annotation and GO classification of the peanut AS genes. The 27,829 AS genes were subjected to GO classification, resulting in 21,499 being assigned to a GO category (Fig. 4). In the “cellular component” category, the genes were concentrated within the groups “cell part” (22.3%), “cell” (22.1%) and “organelle” (19.4%). In the “molecular function” category, the predominant groups were “binding” (38.9%) and “catalytic activity” (42.5%); finally, in the “biological process” category, the genes were distributed quite uniformly between the various groups, with the exception of the under-represented “cell killing process” group. The implication was that AS must be important for the regulation of a wide range of biological processes.

### New genes identification and optimization of the gene structures

Most of the sequences were represented in one or both of the progenitor genomes, while 1776 new genes were not (Additional file 5: Table S5); of these, 1250 were able to be functionally assigned by reference to the various sequence databases (Additional file 5: Table S5). According to COG analysis, a preponderance of these novel genes were concentrated in several categories, such as general function prediction only, replication, recombination and repair, transcription, etc.

The diploid *A. duranensis* and *A. ipaensis* genes (http://www.peanutbase.org) [46] were only annotated using open reading frames (ORFs), and thus most of the 5′- and 3′-untranslated regions (UTRs) have not been defined. Here by globally comparing the complete transcripts with the reference *A. duranensis* and *A. ipaensis* gene models, we successfully elongated the UTRs of 24,717 genes, there into 9177 were elongated with respect to both the 5′- and the 3’-UTR (Additional file 6: Table S6).

### AS related to genes associated with fatty acid metabolism

Acyl-lipid metabolism is a very important process and plays a myriad of diverse functions in all plants. Arabidopsis acyl-lipid metabolism requires more than 600 genes that involved in at least 120 enzymatic reactions. In total, 2275 peanut genes (Additional file 7: Table S7) associated with fatty acid metabolism were identified in the progenitor genomes (excluding transcription factors and ABC transporters) and can be grouped into 12 lipid classes, including fatty acid synthesis and export, plastid glycerolipid synthesis, eukaryotic phospholipid synthesis, etc. Among the 2275 genes, there are 1724 ones with FPKM ≥0.1. More than half of these (1062, accounted for 61.6%) were affected by AS (Additional file 7: Table S7). The high AS ratio indicates the importance of AS in the regulation of fatty acid metabolism. Genes associated with fatty acid synthesis formed the largest group (422 members, of which 226 experienced AS). The 3-ketoacyl-CoA synthase gene family comprised 49 members, but only nine (< 20%) were affected by AS. In contrast, in the acyl-CoA thioesterase gene family, the frequency was 75%.

**Fig. 5:**
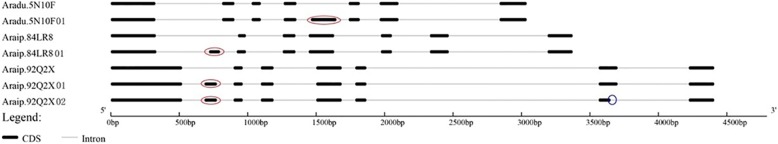
Experimental identification of the AS isoforms of three peanut FAD genes. Red oval indicating the new exons; Blue circle indicating the 3′-AE

Polymerase Chain Reactions (PCR) and sequencings were applied to three genes encoding fatty acid desaturase (FAD) (Aradu.5N10F, Araip.84LR8 and Araip.92Q2X) to validate the presence of AS as predicted from the sequence data (Additional file 4: Table S4). Sequencing of the PCR amplicons established the presence of novel exons among these AS transcripts (Fig. 5). Compared with Aradu.5N10F, Aradu.5N10F01 had gained a new fifth exon; compared with Araip.84LR8, Araip.84LR801 had gained a new second exon; and compared with Araip.92Q2X, Araip.92Q2X01 and Araip.92Q2X02 both gained a new second exon. The difference between Araip.92Q2X01 and Araip.92Q2X02 was due to a 3′-AE event affecting the seventh exon, but these AS events did not influence the translation; all the isoforms could translate into complete protein. By blast these encoded protein sequences in NCBI Nr database we found that they all belonged to FAD gene family.

## Discussion

Since the original discovery that genes can generate multiple transcripts [47], it has become clear that the AS phenomenon is ubiquitous in eukaryote genomes [8, 12, 13, 28–30, 36, 38, 49–51]. The development of high throughput sequencing technologies has allowed for genome-wide scans of AS to be undertaken, resulting in estimates that at least 42% of the intron-containing genes of *A. thaliana* experience AS [31]. According to Zhang et al. [49], the mean number of transcripts generated per gene is 2.4. The proportion of genes which experience AS in plants varied greatly [26]. The proportion of peanut genes thus affected, as estimated here, was about 37.2%, lower than that in tomato, Arabidopsis and soybean [25, 37, 50]. There are several reasons for this: first, the number of the samples we used was less, (seed1, seed2, root and leaf), many genes or AS genes expressed specifically in stem, flower and other tissues were not detected. Many reports showed that AS ratio was related to the sample number, the more samples, the greater the AS ratio [25, 32, 50]. Second, our samples were collected from different developmental stages and did not include samples from different circumstances, so that many genes or AS genes expressed specifically in stress conditions were not detected. It is reported that numerous AS events are induced only by abiotic and biotic stresses [25, 39]. Additionally, the AS ratio discovered within the same tissues and growth conditions is different because of prediction algorithms used [52–54]. In comparison with Arabidopsis and other plants, the study of AS events in peanut was considerably lagged. Here we identified 27,829 peanut genes underwent AS events, about half of the AS genes were constitutively alternatively spliced in all of the four samples, the others showed dramatically differential tissue expression pattern.

Though many AS events regulated by tissue specific cues, it seems that AS plays a particular important role in seed development, for more AS events and AS isoforms were detected in seed than in root and leaf in this study (Tables 1 and 2, Additional file 4: Table S4). Seed is a very important organ of generation containing a mixture of many different tissues. Similar results were also reported in previous researches [25, 50]. Thatcher et al. found that maize seed had more AS isoforms than endosperm and embryo, and there were larger amount of AS isoforms found only in seed [30]. In tomato, the fruits generated more AS isoforms per gene than that of flowers and other organs [50]. Shen et al. found that more AS events occurred in the younger developmental stages than in the older developmental stages for the same type of tissue [25].

It is interesting that more than half of peanut AS genes experienced only a single type of event (Fig. 2a), it means that more peanut AS genes prefer only one AS type to regulate its expression pattern. Potenza et al. reported this phenomenon in grapevine, about 49.7% grapevine AS genes experienced mainly once or twice AS events [51]. It is reported that gene structure has significant influence on AS event types and AS frequency, such as intron length, exon number, gene expression level, etc. [25]. With the increase in the intron length, the proportion of ES increased whereas the proportion of IR decreased; with the increase in the gene expression, the proportion of IR increased, and the proportion of ES decreased [25].

TSS and TTS are commonly detected in different peanut organs. Protein synthesis at the ribosome was directed by the messenger RNA (mRNA) template, so the secondary mRNA structures might influence the translation initiation. Now accumulating researches show that mRNAs could produce protein isoforms owing to the use of TSS, especially in human and mouse [55–59]. In mammals, the TSS transcripts are regulated in a tissue-specific manner and/or developmental stage-specific manner [60]. N-terminomics data shows that in higher eukaryotes around 20% of all identified protein N termini point to such TSS [59]. In plants, studies focusing on TSS have been achieved some progresses. Kitagawa et al. identified many putative TSSs in rice and verified them using Reverse Transcription-Polymerase Chain Reaction (RT–PCR), results showed that TSSs of rice are less diverse than mouse and some of which are regulated in a tissue-specific manner [61]. Another example is the Arabidopsis SYN1 gene, it utilizes alternative promoters and splicing to produce two isoforms with different 5′-ends [62]. Thousands of human and mouse genes generate mRNA isoforms differing in their 3′ UTRs which containing many regulatory elements involved in many cellular processes [63, 64]. Till now, the reason why TTS is so abundant and conserved is still a question. Some research reported that TTS is related to RNA localization, transcript stability and protein production [65], but a recently genome-wide analysis showed acontrary result [66]. TTS could also increase transcription protein diversity. Fontana et al. find a new regulatory mechanism of Brahma (BRM), oxidative stress controls the choice of TTS via a Brahma–BRCA1–CstF pathway [67]. Potenza et al. investigated the location of the AS events in multiple cultivars and found that 86% AS events fall in coding exons, the others occurred in UTR or UTR-CDS [51]. Vitulo et al. found that, in grape, 18 and 11% of all AS events occur at the 5’UTR and 3’UTR regions, and about 1% of the AS events occurred in UTR-CDS [27]. In this study, many TSS and TTS splicing events were identified, and they expressed in a tissue-specific manner (Fig. 5). We think the ratio may be overestimated. The main reason is that most genes in wild type peanut genome were predicted and their UTRs were not identified by experiments, so their UTRs are agnostic. A second reason is that cultivated peanut is allotetraploid, and their genotypic milieu will be more complex than their ancestry. It is reported that whole-genome duplication plays a crucial ploidy-dependent role in AS [28]. These researches indicate that all regions of the transcript are susceptible to AS without any significant preference.

Fatty acid metabolism is a key process in oilseed plants, but little effort has been made to date to define the contribution of AS to this aspect. Thambugala et al. identified six desaturase genes in flax and found some of the SAD and FAD isoforms have significant effects on fatty acid composition, oil content and iodine value [68]. Later, Radovanovic et al. found that all FAD2 isoforms were active, two FAD3A and three FAD3B isoforms were not functional and some of them were caused by the presence of premature stop codon [69]. Here we found that the peanut genome harbors some 1062 genes (FPKM ≥0.1) related to fatty acid synthesis/metabolism, of which around 61.6% were predicted to experience AS; this high proportion suggests that AS likely has a major influence over fatty acid metabolism. Experimental validation of three FADs showed that there indeed exist many AS isoforms in fatty acid metabolism related genes in peanut. But the function of the isoforms needs further study.

## Conclusions

We identified 27,829 AS genes in peanut transcriptome with strand-specific RNA-Seq technique. The occurrence rate of AS genes increased along with the exon number increasing. AS was more frequent in the seed, some of AS genes were organ-specific, the predominant form of AS was intron retention. We analyzed in detail the GO annotation and GO classification of the peanut AS genes. We have cloned some genes to validate the presence of some AS genes as predicted from the sequence data. This identification will have strong impact in the area of peanut study.

## Methods

### RNA isolation, quantification and qualification

Four RNA libraries were developed from a peanut cultivar ‘Fenghua1’: “FH1-seed1” was prepared from seed harvested 30 days after flowering (DAF), “FH1-seed2” from seed harvested after 50 DAF, and both “FH1-root” and “FH1-leaf” from 12 day old seedlings. Each library was based on tissue collected from at least three plants, which was snap-frozen in liquid nitrogen prior to RNA isolation. RNA was extracted using the TRIzol reagent (Invitrogen, Carlsbad, CA, USA) then treated with RNase-free DNase I (New England Biolabs, USA) for 30 min at 37 °C to degrade any contaminating DNA present. The concentration and purity of the resulting RNA preparations were assessed using a NanoDrop 2000 spectrophotometer (Thermo Fisher Scientific, Wilmington, DE, USA) and its integrity was checked using an RNA Nano 6000 Assay Kit (Agilent Technologies, CA, USA).

### cDNA library construction and sequencing

A 1.5 μg aliquot of RNA was processed with a Ribo-Zero rRNA Removal Kit (Epicentre, Madison, WI, USA) to remove the rRNA component, and the subsequently prepared sequencing libraries based on the residual RNA, following treatment with an NEBNext® UltraTM Directional RNA Library Prep Kit for Illumina® (New England Biolabs, USA). Index codes were added to enable each sequence to be attributed its organ provenance. Paired-end sequences were generated by an Illumina Hiseq2500 platform.

### Quantification of gene and transcript abundances and prediction of AS events

After removal of low quality reads and adapter sequences, the sequences were mapped onto the reference peanut genome (https://www.peanutbase.org/) [46] with the aid of TopHat2 software (http://ccb.jhu.edu/software/tophat/index.shtml). The cuffdiff routine within Cufflinks software (http://cufflinks.cbcb.umd.edu/) was used to quantify gene and transcript abundances, based on the FPKM [70]. Transcripts associated with an FPKM greater than 0.1 within a given library were selected as the expression indicator. The overall gene FPKMs were computed by summing the FPKMs of component transcripts. The assembled transcripts were mapped to their corresponding gene model using the Cuffcompare module within the Cufflinks package. AS events was identified using ASprofile software (http://ccb.jhu.edu/software/ASprofile/).

### Functional annotation

Gene function annotation was carried out using a combination of software, tools and databases. InterProScan was used for searching the protein domains and functional sites integrating several different databases (PROSITE, PRINTS, Pfam, ProDom and SMART) [71].The Nr [72], KOG/COG [73], Swiss-Prot [74], KEGG [75] and GO [76] databases were blast at the protein level on the peanut genes.

### Experimental validation of alternative transcripts produced by genes encoding FAD

A 5 μg aliquot of RNA from the FH1-seed1 library was reverse transcribed in a 20 μL reaction using a cDNA synthesis kit (Invitrogen, Carlsbad, CA, USA). Two primers pairs were designed, one targeting Araip.92Q2X and the other Aradu.5N10F and Araip.84LR8. The sequences of the former pair (92Q2X-F/−R) were 5’-TGTGCGTGTTTCATTCACCCTCT/5’-AGGAATTGTGTCATGTGCCTCAT, while those of the latter pair (5N10F-F/−R) were 5’-CATTTTCTCCCACACACTAACTTG and 5’-TGATCATTTAGACTTGTCCGAAG. Each 50 μL RT-PCR contained 100 ng/μL cDNA, 1.5 μL of each primer (10 μM), 5 μL 10 × PCR buffer (Transgen Biotech, Peking, China), 2.5 μL 2.5 mM dNTP and 1 U Trans TaqHiFi DNA polymerase (Transgen Biotech, Peking, China). The reactions were denatured (94 °C/4 min), cycled 30 times through 94 °C/30 s, 58 °C/30 s, 72 °C (120 s), and finally held at 72 °C for 10 min. The PCR products were purified using a MinElute™ Gel Extraction Kit (Qiagen, Hilden, Germany) and inserted into the pEASY-T3 vector (Transgen Biotech, Peking, China) for sequencing. Sequences were compared using DNAMAN software (http://www.lynnon.com/index.html), and gene structures were drawn using Gene Structure Display Server online (http://gsds.cbi.pku.edu.cn/).

## Additional files

Additional file 1: **Table S1.** Sequencing results from the four peanut samples. (DOCX 16 kb)

Additional file 2: **Table S2.** Genome alignment results of the clean data from the four samples. (DOC 31 kb)

Additional file 3: **Table S3.** The number of the isoforms of the AS genes. (XLSX 1826 kb)

Additional file 4: **Table S4.** Expression level of all isoforms. (XLSX 16864 kb)

Additional file 5: **Table S5.** Annotation of the new genes. (XLSX 1294 kb)

Additional file 6: **Table S6.** Optimization of the gene structures. (XLSX 2040 kb)

Additional file 7: **Table S7.** Fatty acid related genes. (XLSX 250 kb)

## Abbreviations

AE: Alternative exon ends
AS: Alternative splicing
BRM: Brahma
DAF: Days after flowering
ES: Exon skipping
FAD: Fatty acid desaturase
FPKM: Fragments per kilobase of exon per million fragments mapped
GO: Gene Ontology
IR: Intron retention
KEGG: Sequences Kyoto Encyclopedia of Genes and Genomes
KOG/COG: Clusters of Orthologous Groups of proteins
MiRNAs: MicroRNAs
mRNA: Messenger RNA
MSKIP: Multiple exon skipping
Nr: Non-redundant protein sequences
ORFs: Open reading frames
PCR: Polymerase Chain Reaction
RT–PCR: Reverse Transcription-Polymerase Chain Reaction
TSS: Transcription start site
TTS: Transcription terminal site
UTRs: 5′- and 3′-untranslated regions
