# Supplementary material for: Transcriptome analysis of alternative splicing in peanut (Arachis hypogaea L.)
Source: BMC Plant Biol. 2018 Jul 4;18:139. doi: 10.1186/s12870-018-1339-9 (PMC6032549; doi:10.1186/s12870-018-1339-9)
Supplement: Supplementary file 1 — Table S1. Sequencing results from the four peanut samples. (DOCX 16 kb) [file 12870_2018_1339_MOESM1_ESM.docx]

Table S1 Sequencing results from the four peanut samples

| Sample ID | Read Sum | Base Sum | GC (%) | N (%) | Q30 (%) |
| --- | --- | --- | --- | --- | --- |
| FH1-seed1 | 55870219 | 14072627030 | 50.48 | 0 | 88.90 |
| FH1-seed2 | 52323196 | 13180643692 | 48.86 | 0 | 89.32 |
| FH1-root | 49291919 | 12416267611 | 47.54 | 0 | 89.59 |
| FH1-leaf | 46359170 | 11674303453 | 44.48 | 0 | 89.79 |

Sample ID：Sample name；Read Sum：Total number of pair-end reads of clean data；Base Sum：total base number of clean data；GC (%)：GC content of clean data (G and C base accounted for a percentage of the total base in clean data); N (%)：the percentage of unresolved bases in total base of clean data；Q30 (%)：Quality value greater than or equal to the percentage of Q30 base in clean data.
