# Supplementary material for: Transcriptome analysis of alternative splicing in peanut (Arachis hypogaea L.)
Source: BMC Plant Biol. 2018 Jul 4;18:139. doi: 10.1186/s12870-018-1339-9 (PMC6032549; doi:10.1186/s12870-018-1339-9)
Supplement: Supplementary file 2 — Table S2. Genome alignment results of the clean data from the four samples. (DOC 31 kb) [file 12870_2018_1339_MOESM2_ESM.doc]

Table S2 Genome alignment results of the clean data from the four samples

| Sample ID | Total Reads | Mapped Reads | Uniq Mapped Reads | Multiple Mapped Reads | Reads Map to '+' | Reads Map to '-' |
| --- | --- | --- | --- | --- | --- | --- |
| FH1-seed1 | 111740438 | 89293748 (79.91%) | 78735194  (88.18%) | 10558554  (11.82%) | 46524610 (41.64%) | 42769138 (38.28%) |
| FH1-seed2 | 104646392 | 86422671 (82.59%) | 73215430  (84.72%) | 13207241  (15.28%) | 44482608 (42.51%) | 41940063 (40.08%) |
| FH1-root | 98583838 | 82965664 (84.16%) | 67101986  (80.88%) | 15863678  (19.12%) | 42559892 (43.17%) | 40405772 (40.99%) |
| FH1-leaf | 92718340 | 81123776 (87.49%) | 53792671  (66.31%) | 27331105  (33.69%) | 41105914 (44.33%) | 40017862 (43.16%) |

Sample ID: Sample name; Total Reads: Number of clean reads (according to the single end gauge); Mapped Reads: The number of reads aligning to the reference genome and the percentage accounting for the clean reads; Uniq Mapped Reads: The number of reads mapping to a reference genome for a single location and the percentage accounting for the clean reads; Multiple Mapped Reads: The number of reads aligning to the reference genome for multiple positions and the percentage accounting for the clean reads: Reads Map to '+': The number of reads aligning to the positive chain of reference genome and the percentage accounting for the clean reads. Reads Map to '-': The number of reads aligning to the negative chain of reference genome and the percentage accounting for the clean reads.
